# FOXP3 and GATA3 Polymorphisms, Vitamin D3 and Multiple Sclerosis

**DOI:** 10.3390/brainsci11040415

**Published:** 2021-03-25

**Authors:** Concetta Scazzone, Luisa Agnello, Bruna Lo Sasso, Giuseppe Salemi, Caterina Maria Gambino, Paolo Ragonese, Giuseppina Candore, Anna Maria Ciaccio, Rosaria Vincenza Giglio, Giulia Bivona, Matteo Vidali, Marcello Ciaccio

**Affiliations:** 1 Department of Biomedicine, Neurosciences and Advanced Diagnostics, Institute of Clinical Biochemistry, Clinical Molecular Medicine and Laboratory Medicine, University of Palermo, 90127 Palermo, Italy; concetta.scazzone@unipa.it (C.S.); luisa.agnello@unipa.it (L.A.); bruna.losasso@unipa.it (B.L.S.); cmgambino@libero.it (C.M.G.); rosaria.vincenza.giglio@alice.it (R.V.G.); giulia.bivona@unipa.it (G.B.); 2 Unit of Neurology, Department of Biomedicine, Neurosciences and Advanced Diagnostics, University of Palermo, 90127 Palermo, Italy; giuseppe.salemi@unipa.it (G.S.); paolo.ragonese@unipa.it (P.R.); 3 Laboratory of Immunopathology and Immunosenescence, Department of Biomedicine, Neurosciences and Advanced Diagnostics, University of Palermo, 90127 Palermo, Italy; giuseppina.candore@unipa.it; 4 Unit of Clinical Biochemistry, University of Palermo, 90127 Palermo, Italy; amciaccio21@gmail.com; 5 Foundation IRCCS Ca’ Granda Ospedale Maggiore Policlinico, 20122 Milan, Italy; matteo.vidali@gmail.com; 6 Department of Laboratory Medicine, Azienda Ospedaliera Universitaria Policlinico “P. Giaccone”, 90127 Palermo, Italy

**Keywords:** multiple sclerosis, genetic, polymorphisms, FOXP3, GATA3, vitamin D

## Abstract

Background: Regulatory T cells (Tregs) alterations have been implicated in the pathogenesis of Multiple Sclerosis (MS). Recently, a crucial role of the X-Linked Forkhead Box P3 (FoxP3) for the development and the stability of Tregs has emerged, and FOXP3 gene polymorphisms have been associated with the susceptibility to autoimmune diseases. The expression of Foxp3 in Tregs is regulated by the transcription factor GATA binding-protein 3 (GATA3) and vitamin D_3_. The aim of this retrospective case-control study was to investigate the potential association between FOXP3 and GATA3 genetic variants, Vitamin D_3_, and MS risk. Methods: We analyzed two polymorphisms in the FOXP3 gene (rs3761547 and rs3761548) and a polymorphism in the GATA3 gene (rs3824662) in 106 MS patients and 113 healthy controls. Serum 25(OH)D3 was also measured in all participants. Results: No statistically significant genotypic and allelic differences were found in the distribution of FOXP3 rs3761547 and rs3761548, or GATA3 rs3824662 in the MS patients, compared with controls. Patients that were homozygous for rs3761547 had lower 25(OH)D3 levels. Conclusions: Our findings did not show any association among FOXP3 and GATA3 SNPs, vitamin D_3_, and MS susceptibility.

## 1. Introduction

Multiple Sclerosis (MS) is a chronic autoimmune inflammatory disease of the central nervous system (CNS). Studies on Experimental Autoimmune Encephalomyelitis (EAE), which represents the best animal model of MS, made a significant contribution to the understanding of MS pathogenesis. It is now well documented that CD4 (+) and CD8 (+) T lymphocytes and their related cytokines, as well as B-lymphocytes, take part in the development of MS. Among CD4 (+) T cells, it is possible to distinguish different cell subsets according to their cytokine secretion pattern [1]. Specifically, Th1 and Th17 cells produce pro-inflammatory cytokines, such as IFN-γ and IL-17, respectively, whereas Th2 and regulatory T cells (Tregs) produce anti-inflammatory cytokines, such as IL-10 [2,3]. Tregs have an essential role in controlling the immune system by several mechanisms, including regulation of antigen-presenting cells (APC) function, induction of tolerance, cytolysis, and expression of inhibitory cytokines [4]. Overall, Tregs are fundamental in maintaining immune self-tolerance and immune homeostasis, limiting excessive inflammation. Alterations of Tregs, including numerical reduction and functional changes, have been implied in the immune-mediated damage of myelin and axons, leading to neuronal damage and neuroinflammation in MS [5]. Moreover, reduced migration of Tregs into CNS of MS patients has been described [6]. Noteworthy, master-regulators of transcription are essential for T lymphocytes function [1]. Among these, the X-Linked Forkhead Box P3 (FoxP3) has a crucial role in Tregs development and stability, as shown by in vivo and in vitro studies [7,8,9]. In particular, FOXP3-deficient Treg cells have been shown to reduce expression of Treg cell signature genes, such as TGF-β, IL-10, and CTLA4, which are critical for tolerance and immunosuppression, while gained the expression of cytokine genes, such as IFN-γ, TNF-α, and IL-17, which stimulate the immune response [7]. Many polymorphisms in the gene codifying for Foxp3 have been associated with reduced levels of Foxp3 and impaired suppressive function of Treg cells, resulting in the development of autoimmune diseases [10]. An association between single nucleotide polymorphisms (SNPs) of the FOXP3 gene and autoimmune diseases, such as allergy, Graves’ disease, and systemic lupus erythematosus, has been described [11,12,13].

Additionally, the sustained trek expression of Foxp3 is regulated by several factors, including the transcription factor GATA binding-protein 3 (GATA3) and vitamin D_3_. In vivo and in vitro studies showed that GATA3 expression has a fundamental role in maintaining high-levels of Foxp3 in Tregs [14]. GATA3 has been reported to prevent excessive polarization toward Th17 and inflammatory cytokine production of Treg cells. Indeed, GATA-3-null Treg cells have been shown to fail to maintain peripheral homeostasis and suppressive function, shifting toward Th17 cell phenotypes and expressing reduced amounts of Foxp3 [15].

Vitamin D_3_ has a pivotal role in regulating the immune system [16,17,18,19]. An association between reduced levels of vitamin D_3_ and increased risk of several autoimmune diseases, including MS, has been documented. Several hypotheses have been proposed to explain the potential role of vitamin D in the pathophysiology of MS. Among these, experimental studies revealed that 1,25-dihydroxivitamin D3 (1,25(OH)_2_D_3_) regulates FOXP3 expression in Tregs [20]. Thus, reduced vitamin D_3_ levels could be associated with reduced FOXP3 expression and, consequently, could increase the risk of MS.

The aim of this study was to investigate the association among SNPs in FOXP3 and GATA3 genes, vitamin D_3_, and MS susceptibility. Specifically, we selected two SNPs in FOXP3 gene, namely rs3761547 and rs3761548, and the rs3824662 in the GATA3 gene, which could influence the FOXP3 and GATA3 expression, respectively. Thus, they could predispose to the development of autoimmune diseases, such as MS.

## 2. Materials and Methods

### 2.1. Study Population

We performed a retrospective case-control study on a cohort consisting of 106 patients with MS and 113 healthy controls. Cases were enrolled from June 2013 to December 2014 at the Department of Neurology, University Hospital of Palermo. Healthy controls were blood donors, enrolled from April 2015 to July 2016, at the Unit of Transfusion Medicine of Villa Sofia-Cervello Hospital in Palermo. The study was performed in accordance with the Declaration of Helsinki, and the local medical ethics committee approved the protocol. All subjects provided informed consent. An experienced neurologist made the diagnosis of MS according to revised McDonald criteria [21]. The neurological status of patients was assessed using Kurtzke’s Expanded Disability Status Scale (EDSS). The progression of disability was assessed using the Multiple Sclerosis Severity Score (MSSS) [22]. The annualized relapse rate (ARR) was calculated in the year before genotyping. The MS group consisted of 27 men and 79 women, median (IQR) age 39 (34–48) years. Eighty-four percent of patients were diagnosed with the relapsing-remitting form of the disease (RRMS), 15% with the secondary-progressive form (SPMS), and 1% with the primary progressive form (PPMS). The overall median (IQR) of disease duration was 10.6 (2.4–20.2), EDSS score 2.3 (1.4–5.0), MSSS score 3.3 (1.5–5.5), and ARR scores 1 (1–2).

The control group consisted of 58 men and 55 women with a median (IQR) age of 40 years (28–49).

The study protocol was approved by the Ethics Committee of the University Hospital of Palermo (nr 07/2016) and was performed in accordance with the current revision of the Helsinki Declaration. Informed consent was obtained from all individual participants included in the study.

### 2.2. Molecular Analysis

Whole blood samples from patients and controls were collected in EDTA tubes and stored at 4 °C for subsequent DNA extraction. Genomic DNA was extracted from 200 μL of whole blood using a commercial kit (Qiagen, Valencia, CA, USA), according to the manufacturer’s instructions. The DNA quality was evaluated by electrophoresis in a 0.8% agarose gel, quantified by using absorbance spectrophotometric analysis and stored at −20 °C for subsequent analysis.

We selected two SNPs in the FOXP3 gene, namely rs3761548 and rs3761547, and a SNP in the GATA3 gene, namely rs3824662, based on evidence in the literature [23,24]. Characteristics of all selected SNPs are shown in Table 1. We used the following primers (VIC/FAM): TGTCTGCAGGGCTTCAAGTTGACAA(T/C)TGCCCCTCTATCCAGGGGACTGGCT for rs3761547; GGTGCTGAGGGGTAAACTGAGGCCT(T/G)CAGTTGGGGAGAGAGCCAGAACCAG for rs3761548; AGGAAGGCGCCTTTGGCATGCACTG(A/C)AGCGTGTTTGTGTTTAATCTCAGGG for rs3824662.

All samples were genotyped using real-time allelic discrimination TaqMan assay (Applied Biosystems). The genotyping was performed by a 7500 real-time PCR system. All PCR reaction mixtures contained 1 μL DNA (≈25 ng), 5 μL TaqMan Genotyping Master Mix, and 0.25 μL genotyping Assay mix containing primers and FAM- or VIC-labeled probes and distilled water for a final volume of 20 μL. The real-time PCR conditions were initially 60 °C for 30 s and then 95 °C for 10 min, and subsequently 40 cycles of amplification (95 °C for 15 s and 60 °C for 1 min), and finally 60 °C for 30 s (Applied Biosystems).

### 2.3. Biochemical Analysis

Serum 25(OH)D_3_ levels were measured by high-performance liquid chromatography (HPLC) using a Chromosystem reagent kit (Chromsystems Instruments & Chemicals GmbH; Grafelfing, Munich, Germany).

According to the recommendation of the Institute of Medicine, vitamin D_3_ deficiency was defined as serum 25(OH)D_3_ < 20 ng/mL, vitamin D_3_ insufficiency as serum 25(OH)D_3_ levels 20–30 ng/mL, and vitamin D_3_ sufficiency as serum 25(OH)D_3_ > 30 ng/mL.

### 2.4. Statistical Analysis

Statistical analysis was performed by SPSS version 17.0 (SPSS Inc., Chicago, IL, USA) and R Language v.3.6.1 (R Foundation for Statistical Computing, Vienna, Austria). Quantitative variables were expressed by the median and interquartile range (IQR) while categorical variables by absolute and relative frequencies. All genotypes were tested for Hardy–Weinberg equilibrium by using an exact test. Differences in age or vitamin D levels between MS patients and controls were evaluated by both parametric *t*-test and non-parametric Mann–Whitney test. The association between MS diagnosis (dependent dichotomous variable) or vitamin D levels (continuous dependent variable) and predictors was evaluated, respectively, by multivariate logistic regression and General Linear Model. Association with chrX SNP was evaluated assuming (0, 2) dosage for males and adjusting for sex.

## 3. Results

We enrolled 106 MS patients and 113 healthy controls. Table 2 shows the clinical characteristics of the study population. The polymorphisms were in Hardy–Weinberg equilibrium (*p* > 0.05). Genotype and allele frequencies of cases and controls are shown in Table 3 and Table 4. No significant statistical association was found by logistic regression between FOXP3 rs3761547 and rs3761548 as well as GATA3 rs3824662 genotypes and MS disease. Moreover, no association was found between FOXP3 rs3761548 and rs3761547 or GATA3 rs3824662 genotypes on the age of disease onset (*p* ranging from 0.284 to 0.955), diseases duration (*p* ranging from 0.259 to 0.547) EDSS (*p* ranging from 0.631 to 0.985), MSSS (*p* ranging from 0.601 to 0.680) and ARR (p ranging from 0.203 to 0.900).

We found that serum 25(OH)D_3_ levels were significantly lower in MS patients than in controls (20.0 (15.0–25.0)μg/L and 39.0 (28.5–49.0)μg/L, respectively; *p* < 0.001). In particular, vitamin D_3_ insufficiency was prevalent in MS patients (59%); 26% had vitamin D_3_ deficiency, and only 15% had optimal levels. Moreover, men in the whole sample displayed significantly higher levels of vitamin D_3_ than women (median 33 (24–45) μg/L vs. 25 (19–35) μg/L; *p* = 0.007). Nevertheless, vitamin D_3_ was not associated with age (*p* = 0.683).

Multivariate analysis was performed using vitamin D_3_ levels as a dependent variable, while age, sex, MS diagnosis, and studied polymorphisms (three different models) as independent variables. The analysis showed that none of the three studied polymorphisms were associated with vitamin D levels (*p* ranging from 0.270 to 0.894). Interestingly, the only independent predictor that was found significantly associated with vitamin D_3_ levels in all three models investigated was the presence of the MS, further supporting previous literature results on the role of vitamin D in MS.

## 4. Discussion

MS is a multifactorial disease that occurs in genetically susceptible individuals after exposure to environmental factors, which contribute to the loss of tolerance and activation of T cells to myelin antigens [26,27].

Genetic studies uncovered several gene variants, which are putatively associated with MS susceptibility, including those codifying molecules involved in vitamin D_3_ metabolism [28,29,30,31,32,33]. However, an essential role for Foxp3 has recently emerged. Foxp3 is a transcription factor belonging to the forkhead/winged-helix transcription factor family, and it is fundamental for maintaining the suppressive activity of Tregs [34,35]. Genetic variants of FOXP3 have been associated with an impaired function and differentiation of Treg cells, resulting in autoimmune dysfunction. Some Authors reported altered expression of Foxp3 in patients with MS [36,37,38]. A transcriptional factor critical for Treg cell function and Foxp3 expression is GATA3 [39], as revealed by in vivo studies [15]. Wang et al. [15] showed that GATA-3-null Treg cells failed to maintain peripheral homeostasis and suppressive function, gained Th17 cell phenotypes, and expressed reduced Foxp3 levels. Finally, vitamin D_3_ has an important role in regulating the expression of FOXP3.

Given this evidence, we performed a case-control study to evaluate the possible influence of two SNPs in the promoter region of FOXP3 and an SNP of GATA3 on genetic predisposition to MS. Moreover, we investigated the relationship between such polymorphisms and 25(OH)D_3_.

Among the selected FOXP3 SNPs, a functional effect has been reported only for the rs3761548 [25]. In particular, the minor allele A has been associated with an impaired interaction of the promoter region of FOXP3 with transcription factors, such as E47 and C-Myb, which reduce FOXP3 transcription. Similarly, the rs3824662 GATA3 has been associated with altered GATA3 expression [23].

In this study, we did not find any association among the selected SNPs, MS susceptibility, and 25(OH)D_3_. Although the influence of FOXP3 SNPs on disease risk has been established in several autoimmune diseases, such as systemic sclerosis [40] and asthma [41], contrasting results have been reported in MS patients. Jafarzadeh et al. [42] and Eftekharian et al. [43] found an association between rs3761548 FOXP3 gene and MS in an Iranian population. Recently, Wawrusiewicz-Kurylonek et al. [24] investigated the association of three SNPs of FOXP3, including the rs3761548 and the rs3761547, on MS risk in a Polish population. The authors found a gender-specific relation between rs3761547 FOXP3 gene polymorphism and MS susceptibility. In particular, such polymorphism was associated with increased risk only in male patients. On the other hand, Gajdošechová et al. failed to find any association between FOXP3 SNPs and MS risk in a Slovak population [44]. Similar results were found by Işik et al. [45]. Flauzino et al. found an association between the rs3761548 FOXP3 and MS in females in a Brazilian population [46]. Additionally, Zhang et al. [47] performed a meta-analysis on five studies investigating the correlation between FOXP3 polymorphisms and MS risk. Authors showed that the rs3761548 could be associated with MS susceptibility, especially in Asians. However, such meta-analysis has several biases, including the low number of studies included. Additionally, four out of five studies were performed on Asians. Thus, the results may do not apply to different ethnic populations, such as Europeans. Indeed, it is well recognized that ethnicity affects the genetic background, resulting in contrasting results among different populations. The inconsistency of the results among the different studies on the association of FOXP3 SNPs and MS could be due to several reasons, including the sample size, the genotyping methods (restriction fragment length polymorphism, real-time PCR), and the selection of controls (hospital-based controls, community-based controls, healthy blood donors) [48]. To the best of our knowledge, this is the first study evaluating the role of FOXP3 polymorphisms on MS risk in an Italian population and the first investigating the possible influence of GATA3 polymorphism in MS.

Limitations of this study are small sample size, case-control design, the lack of match between cases and controls, the lack of sample size estimation prior to starting the study, and the lack of assessment of cytokines in order to characterize better the complex relationship among FOXP3, chemical mediators and MS.

## 5. Conclusions

It is sound to investigate FOXP3, and GATA3 SNPs within the contest of immune-mediated diseases as MS since functional alterations of these proteins could be involved in the development of such diseases. FOXP3 and GATA3 exert immune suppressive activity; however, some of their gene variants have been shown to impair immune-suppressive activity, thus contributing to the development of autoimmune diseases. In this case-control study, we evaluated the possible role of two SNPs of the FOXP3 gene, the rs3761547 and rs3761548, and an SNP of the GATA3 gene, rs3824662, as a susceptibility risk factor for MS. We did not find an association between the selected SNPs and MS risk. However, it is possible that several factors, including the small sample size, could have influenced our results. Therefore, we cannot draw final conclusions on the role of GATA3 and FOXP3 polymorphisms on the MS risk. Accordingly, it is worth studying FOXP3 and GATA3 SNPs in larger populations to understand better whether they could have a role in MS susceptibility.

## Figures and Tables

**Table 1:** Characteristics of FOXP3 and GATA3 single nucleotide polymorphisms (SNPs).

Gene	Chromosome	SNP	Ancestral Allele	Substitution Allele	SNP Location	Functional Effect	Ref.
FOXP3	X	rs3761547	T	C	Intron	Unknown	-
		rs3761548	G	T	Intron	Reduced expression	[25]
GATA3	10	rs3824662	C	A	Intron	Altered expression	[23]

**Table 2:** Demographic and clinical characteristics of multiple sclerosis (MS) patients and controls.

	MS (*n* = 106)	Controls (*n* = 113)	*p*-Value
Age (years)	39 (34–48)	40 (28–49)	0.703
Sex, n (male/female)	27/79	58/55	<0.001
25(OH)_3_, μg/L	20.0 (15.0–25.0)	39.0 (28.5–49.0)	<0.001
Disease duration (years)	10.6 (2.4–20.2)	-	
Age of MS onset (years)	28 (22–32)	-	
MS-type (%) RR/SP/PP	84/15/1	-	
EDSS	2.3 (1.4–5)	-	
MSSS	3.3 (1.5–5.5)	-	
ARR	1 (1–2)	-	

Data are shown as: median (interquartile range), RR, Relapsing Remitting; SP, Secodary Progressive; PP, Primary Progressive; EDSS = Expanded Disability Status Scale; MSSS = Multiple Sclerosis Severity Score; ARR = annualized relapse rate.

**Table 3:** Distribution of genotypic and allelic frequencies of the FOXP3 SNPs in MS patients and controls.

SNP	Patients (*n*/%)		Controls (*n*/%)		*p*-Value
rs3761548 (FOXP3)	Male (27)	Female (79)	Male (58)	Female (55)	
GG		12 (15)		11 (21)	0.997
TG		41 (52)		30 (54)	
TT		26 (33)		14 (25)	
G	15 (56)		26 (45)		
T	12 (44)		32 (55)		
rs3761547 (FOXP3)	Male (27)	Female (79)	Male (58)	Female (55)	
TT		63 (80)		44 (80)	0.460
TC		14 (18)		9 (16)	
CC		2 (2)		2 (4)	
T	24 (88)		56 (97)		
C	3 (12)		2 (3)		

**Table 4:** Distribution of genotypic and allelic frequencies of the GATA3 SNP in MS patients and controls.

SNP	Patients (n/%)	Controls (n/%)	*p*-Value
rs3824662 (GATA3)			
CC	61 (57)	64 (54)	0.945
CA	39(37)	43 (38)	
AA	6 (6)	6 (8)	
C	161 (76)	171 (75)	
A	51 (24)	55 (25)	

## Data Availability

The data that support the findings of this study are available from the corresponding author upon reasonable request.
